# The stem cell organisation, and the proliferative and gene expression profile of Barrett's epithelium, replicates pyloric-type gastric glands

**DOI:** 10.1136/gutjnl-2013-306508

**Published:** 2014-02-18

**Authors:** Danielle L Lavery, Anna M Nicholson, Richard Poulsom, Rosemary Jeffery, Alia Hussain, Laura J Gay, Janusz A Jankowski, Sebastian S Zeki, Hugh Barr, Rebecca Harrison, James Going, Sritharan Kadirkamanathan, Peter Davis, Timothy Underwood, Marco R Novelli, Manuel Rodriguez–Justo, Neil Shepherd, Marnix Jansen, Nicholas A Wright, Stuart A C McDonald

**Affiliations:** 1 Epithelial Stem Cell Group, Centre for Tumour Biology, Barts Cancer Institute, Barts and The London School of Medicine and Dentistry, Queen Mary University of London, London, UK; 2 Stem Cell Biology of the Intestine Laboratory, Cancer Research UK Cambridge Research Institute, Li Ka Shing Centre, Cambridge, UK; 3 Centre for Digestive Diseases, Blizard Institute, Barts and the London School of Medicine and Dentistry, Queen Mary University of London, London, UK; 4 Department of Surgery, Gloucestershire Royal Hospital, Gloucestershire Royal Hospital, Gloucester, UK; 5 Department of Pathology, University Hospitals Leicester, Leicester, UK; 6 University of Glasgow, Institute of Cancer Sciences, Glasgow, UK; 7 Mid Essex Hospital Services NHS Trust, Broomfield Hospital, Chelmsford, UK; 8 Faculty of Medicine, University of Southampton, Southampton, UK; 9 Department of Histopathology, University College London, London, UK; 10 Gloucestershire Cellular Pathology Laboratory, Cheltenham General Hospital, Cheltenham, UK; 11 Department of Pathology, Academic Medical Center (AMC), Amsterdam, The Netherlands

**Keywords:** Barrett's Oesophagus, Gene Expression, Stem Cells, Trefoil Factors, Mucins

## Abstract

**Objective:**

Barrett's oesophagus shows appearances described as ‘intestinal metaplasia’, in structures called ‘crypts’ but do not typically display crypt architecture. Here, we investigate their relationship to gastric glands.

**Methods:**

Cell proliferation and migration within Barrett's glands was assessed by Ki67 and iododeoxyuridine (IdU) labelling. Expression of mucin core proteins (MUC), trefoil family factor (TFF) peptides and *LGR5* mRNA was determined by immunohistochemistry or by in situ hybridisation, and clonality was elucidated using mitochondrial DNA (mtDNA) mutations combined with mucin histochemistry.

**Results:**

Proliferation predominantly occurs in the middle of Barrett's glands, diminishing towards the surface and the base: IdU dynamics demonstrate bidirectional migration, similar to gastric glands. Distribution of MUC5AC, TFF1, MUC6 and TFF2 in Barrett's mirrors pyloric glands and is preserved in Barrett's dysplasia. MUC2-positive goblet cells are localised above the neck in Barrett's glands, and TFF3 is concentrated in the same region. *LGR5* mRNA is detected in the middle of Barrett's glands suggesting a stem cell niche in this locale, similar to that in the gastric pylorus, and distinct from gastric intestinal metaplasia. Gastric and intestinal cell lineages within Barrett's glands are clonal, indicating derivation from a single stem cell.

**Conclusions:**

Barrett's shows the proliferative and stem cell architecture, and pattern of gene expression of pyloric gastric glands, maintained by stem cells showing gastric and intestinal differentiation: neutral drift may suggest that intestinal differentiation advances with time, a concept critical for the understanding of the origin and development of Barrett's oesophagus.

**Significance of this study**

**What is already known on this subject?**

- Barrett's glands are protean and contain a wide range of differentiated cell lineages.
- Barrett's glands are clonal and contain multiple, multipotent stem cells.
- Dogma states that Barrett's is a metaplasia of the squamous oesophagus.

**What are the new findings?**

- The gene expression and proliferative compartments of Barrett's glands reflects that of pyloric glands which also demonstrate bidirectional migration, as seen in gastric glands.
- The stem cell zone is located at the neck of Barrett's glands, similar to pyloric glands: gastric and intestinal cell lineages within Barrett's glands show a common stem cell origin.
- ‘Specialised’ gastric glands resemble pyloric glands showing partial intestinalisation.
- The gastric gland architecture and organisation is maintained in dysplasia.

**How might it impact on clinical practice in the foreseeable future?**

- Endoscopic and pathological examination of the gastric cardia may need to be more rigorous in gastro-oesophageal reflux disease patients.
- More investigation into determining glandular phenotype as a biomarker of progression to dysplasia in Barrett's patients is needed.
- Any unifying proposal for the origin and development of Barrett's oesophagus should explain their resemblance to pyloric glands.

## Introduction

Barrett's oesophagus remains an enigma with no agreement about its origin,^1^^–^3 and the nature of the epithelium in Barrett's mucosa is disputed,^4^ ^5^ with controversy regarding which epithelial component progresses to cancer. The belief that intestinal metaplasia (or ‘specialised epithelium’), where goblet cells are plentiful, is typically required for the diagnosis of Barrett's oesophagus^2^ has lead to Barrett's mucosa being regarded as ‘intestinal metaplasia’, and the mucosal units as ‘crypts’, resembling crypts in the intestine. However, the phenotype of Barrett's oesophagus is *protean* containing a variety of cell lineages. Even in ‘specialised epithelium’, there are *multiple* cell lineages: columnar cells resembling gastric foveolar cells containing MUC1, MUC5AC and mucus secreting cells expressing MUC6—mucin core proteins characteristic of gastric epithelium,^6^ ^7^ and goblet cells, with MUC2 and MUC3—seen in intestinal epithelium.^8^ Thus, the so-called ‘specialised epithelium’ of Barrett's oesophagus, often compared with intestinal metaplasia, shows evidence of *gastric lineage differentiation* as well as intestinal differentiation.

Barrett's mucosa contains several different types of glands—Paull *et al*,^9^ by mapping the distribution of the several phenotypes, reported a *zonal distribution* of the different types of mucosa, with oxyntic-type glands with parietal and chief cells or oxynto-cardiac glands interposed between the specialised columnar epithelium and the lower oesophageal sphincter. Such ‘zonation’ has been replicated, although some reports^10^ ^11^ have found the different phenotypes randomly distributed throughout Barrett's mucosa. There is a gradient of goblet cell density, with significantly lower numbers seen in the distal Barrett’s segment,^10^ correlated with an oesophageal luminal pH gradient.^11^ Cardiac mucosa is present throughout the segment, with oxynto-cardiac mucosa more frequently found distally.^9^ ^10^ Going *et al*,^10^ reported higher frequencies of cardiac and oxynto-cardiac mucosa in the distal Barrett's segment, with several different mucosal phenotypes at any single anatomical level, although ‘specialised’ epithelium was found at all levels. Glands showing intestinalisation, resembling ‘complete type’ (Type I) intestinal metaplasia, characterised by the presence of absorptive cells, Paneth cells, and goblet cells, are also seen.^10^ Thus, Barrett's oesophagus contains multiple lineages and glands with several phenotypes.

Human intestinal crypts and gastric glands are *clonal populations*—derived from a single tissue-specific stem cell.^12^ These clonal crypts and glands contain multiple, multipotential stem cells from which all the contained lineages derive: studies using mitochondrial DNA (mtDNA) mutations that cause cytochrome *c* oxidase (CCO) deficiency as clonal markers, showed Barrett’s metaplastic glands as clonal units maintained by multiple stem cells, and all epithelial cell lineages within a gland derived from multipotential stem cells.^13^ Thus, whatever the complexity of a Barrett's gland, whatever heterogeneous cell lineages it contains, it was derived from *a single stem cell and that original stem cell's progeny has sufficient multipotentiality to maintain its multilineage habitus*.

Barrett's glands show evidence of gastric and intestinal differentiation patterns: if such glands are clonal, the stem cell(s) will show capacity to differentiate into gastric and intestinal cell lineages. We show that *specialised* Barrett's glands show maximal proliferation in the middle part of the gland, that cells migrate in a bidirectional manner and that the stem cell niche is located in the middle part of the gland, resembling the gastric gland and not the intestinal crypt. Region-specific gene expression supports a gastric gland plan, and we propose that Barrett's glands are maintained by stem cells with gastric and intestinal differentiation capacity that progress to intestinal type over time.

## Materials and methods

*Tissues*: formalin-fixed, paraffin-embedded archival non-dysplastic Barrett's oesophagus and Barrett's dysplasia tissue specimens and frozen specimens were obtained from patients undergoing oesophagectomy or endoscopic mucosal resection for adenocarcinoma or dysplasia (n=34). Normal gastric and intestinal metaplastic formalin-fixed, paraffin-embedded specimens were obtained from patients undergoing resection for either gastric carcinoma or high-grade dysplasia (n=23). Histological examination following standard H&E staining and periodic acid Schiff/Alcian blue staining was carried out by at least two qualified pathologists (RH, MR-J, MRN, NS or NAW). Ethical approval was sought and obtained from the London research ethics committee, Stanmore11/LO/1613.

**Figure 1:**
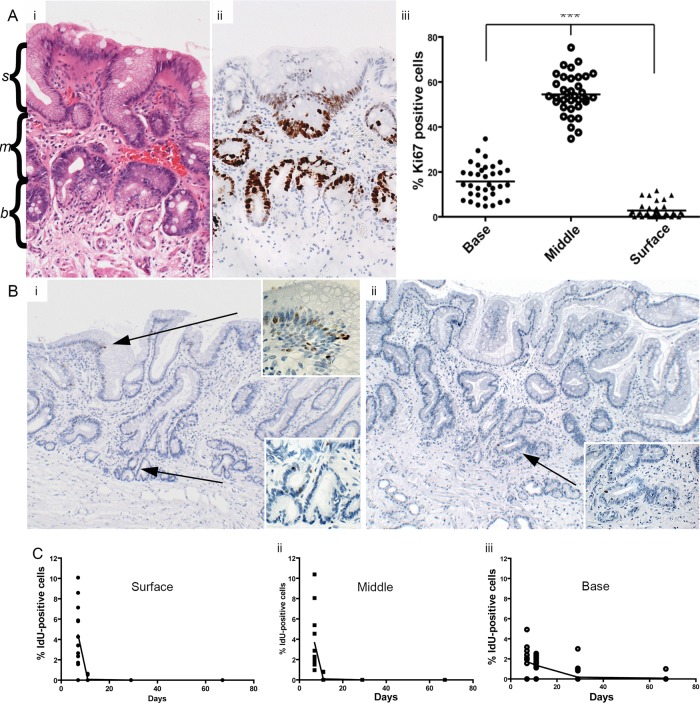
(A) (i) H&E (highlighted with *s*(surface), *m*(middle) and *b*(base)) and (ii) showing Ki67 expression in Barrett's glands; (iii) The number of Ki67+ cells in each region of Barrett's glands; (B) (i) IdU+ cells in the base, middle and surface of Barrett's glands 7 days. Inserts show high-power images of IdU+ cells; (ii) IdU+ cells at 11 days (arrowed). Inserts show a high-power image of IdU+ cells. (C) The changes in the distribution of IdU+ cells Barrett's glands with time after IdU injection. (i) IdU+ cells within the foveolus of the gland rapidly disappear and cannot be identified after 11 days; (ii) IdU+ cells identified within the middle of the gland decrease more rapidly after 11 days; (iii) the incidence of IdU+ cells in the base of Barrett's glands falls slowly up to 67 days after infusion.

*Immunohistochemistry (IHC)* was carried out using methods described in online supplementary methods. The numbers of Ki67+ and IdU+ cells were scored within Barrett's glands as follows: two tissue sections from each of the patients were included and three areas of approximately 100 cells were scored per section. For cell counts, glands were divided into three equal regions: the bottom third was designated the gland base-corresponding to the Muc6+/trefoil family factor 2 (TFF2)+ mucus secreting zone, and the remaining upper two-thirds of the gland were divided equally and designated the middle region and the surface of the gland, respectively (highlighted in figure 1A).

In situ *hybridisation* (ISH) was carried out using the methods described in online supplementary methods.

*Clinical protocol for labelling with iododeoxyuridine (IdU)*: The Stem Cell Assessment In Neoplastic Tissues (SAINT) trial (n=4 patients) was approved by the Leicestershire Ethics Board Ref Number: 09122, Medicines Health Regulatory Authority Number: CTA 21275 and Research Ethics Committee Number: 7213 as previously described.^14^ Prior to oesophagectomy, oesophageal Barrett's adenocarcinoma patients were infused with IdU, and details are given in online supplementary methods.

*Laser capture microdissection and PCR sequencing of MtDNA*: This was carried out using the methods described in online supplementary methods.

## Results

### Proliferative organisation in Barrett's glands

Ki67+ cells were concentrated in the middle region of Barrett's gland with fewer proliferating cells at the surface of the glands, little at the bases and none in the very basal cells (figure 1A, and online supplementary figure S1). This pattern of expression was consistent throughout all study patients. The majority of Ki67+ cells were seen within the middle region of the gland in all patients (54.5% of total cells). The percentage of Ki67+ cells within the middle region was shown to be significantly greater than that of the base of the gland and the gland surface within these patients (Kruskal-Wallis one-way analysis of variance, p<0.05).

### Cell migration in Barrett's glands

At 7 days postinjection, IdU+ cells were seen towards the base and middle of the gland and also at the surface (figure 1B), clustered together in these gland segments. At 11 days, IdU+ cells were seen in the base of the gland, in the middle and also at the surface of the gland, but the number of IdU+ cells was reduced compared to 7 days with the majority of positive cells seen towards the gland bases (figure 1B) suggesting that the superficial labelled cells have been rapidly lost into the lumen, with only a few still migrating, but that migration towards the base is slower. At 29 days after injection, IdU+ cells were still evident, although greatly reduced compared to 11 days, and were seen almost exclusively in the bases of the glands, where a few cells were still evident at 67 days after injection^1^.^4^

The distribution of IdU+ cells within Barrett's glands over time was quantified as described above: figure 1C shows the distribution of IdU+ cells in Barrett's glands with time after IdU injection. After 7 days, 4.45% of IdU+ cells were observed within the surface of the gland, although labelled cells were still numerous within the middle region (3.65%). However, fewer IdU+ cells were observed within the gland base (1.74%). After 11 days, IdU+ cells were still observed within the surface of the glands, although the fraction had significantly reduced and they now represented 0.07% of total cells. The highest percentage was observed within the base of the Barrett's gland (1.36%). After 29 days chase, the number of IdU+ cells was significantly reduced compared to 11 days, and were exclusively observed within the base of the Barrett's glands (0.181% of total cells). Furthermore, the number was reduced again after 67 days chase: at this time IdU+ cells were seen exclusively within the base of the gland (0.07%).

Taken together with the Ki67 labelling index distribution, which shows that the bases of Barrett's glands contain few labelled cells (see online supplementary figure S1), and since maximal cell proliferation is present in the middle of the gland, cells migrate faster towards the surface or foveolar portion of the gland, and slowly into the base of the gland. Labelled cells in Barrett's glands show *bidirectional flux*.

In corpus gastric glands, one tissue section from each patient was included and three areas of over 100 cells within each region of the gastric unit (foveolus or pit, isthmus/neck and gland base) and were scored per slide. Online supplementary figures S2A,B show that Ki67-labelled cells occur mainly within the neck/isthmus regions of the gastric unit (15.2% of total cells) with fewer Ki67+ cells observed within the foveolus (9.5%) and 1.5% in the gland base. A similar distribution is shown in gastric antral mucosa (see online supplementary figure S2B). After 7 days, the highest percentage of IdU+ cells was observed within the foveolar region (3.3%) (see online supplementary figures S2C,D), whereas at 11 days, the highest percentage of cells was observed within the neck (0.27%) (see online supplementary figures S2E,F). There was a significant reduction in IdU+ cells within the neck and the foveolar regions between 7 and 11 days, suggesting that cellular flux occurs mainly in the foveolus, since 7 days postinjection most IdU+ cells are identified within this region. Most cells are lost into the lumen after 11 days, yet some cells remain within the neck region of the gland. However, IdU-labelled parietal cells were seen towards the bases of gastric glands at 67 days after infusion of IdU^14^.

Cell flux is bidirectional in Barrett's glands, similar to that seen in gastric glands.

### The stem cell niche in Barrett's glands

**Figure 2:**
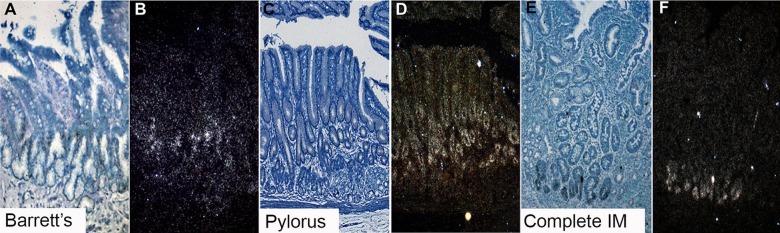
*LGR5* mRNA expression using in situ *hybridisation*. (A, B) A bright field image and accompanying dark field image of *LGR5* mRNA in Barrett's glands; (C and D) A bright field image and accompanying dark field image of *LGR5* mRNA of pyloric gastric glands; (E and F) A bright field image and accompanying dark field image of *LGR5* mRNA in gastric intestinal metaplasia.

Here, stem cells were identified by ISH for the established gastrointestinal epithelial stem cell marker LGR5.^15^ Figure 2 shows the distribution of cells which express *LGR5* mRNA in Barrett's glands (A, B), in pyloric glands (C, D) and in the crypts of gastric intestinal metaplasia (C, F). Figures are representative of n=5. In the pyloric glands (figure 2C,D) *LGR5* mRNA is seen quite widely distributed in the isthmus/neck area of the glands, while the foveola and the mucin-secreting bases of the glands are negative. In Barrett's glands (figure 2A,B) *LGR5* mRNA is localised in the middle of the gland, corresponding to the equivalent of the isthmus/pit in a pyloric gland. Figures 2E and F show that in intestinal metaplasia in the stomach, *LGR5* mRNA is found at the bases of the crypts, similar to colonic crypts (see online supplementary figure S3).

The stem cell niche in Barrett's oesophagus is localised in the middle of the gland, in the equivalent of the isthmus/pit of a pyloric gland, as seen in pyloric gastric glands. In gastric intestinal metaplasia, the stem cell niche is at the base of the crypt, comparable with the colon.

### Clonal organisation of Barrett's glands

**Figure 3:**
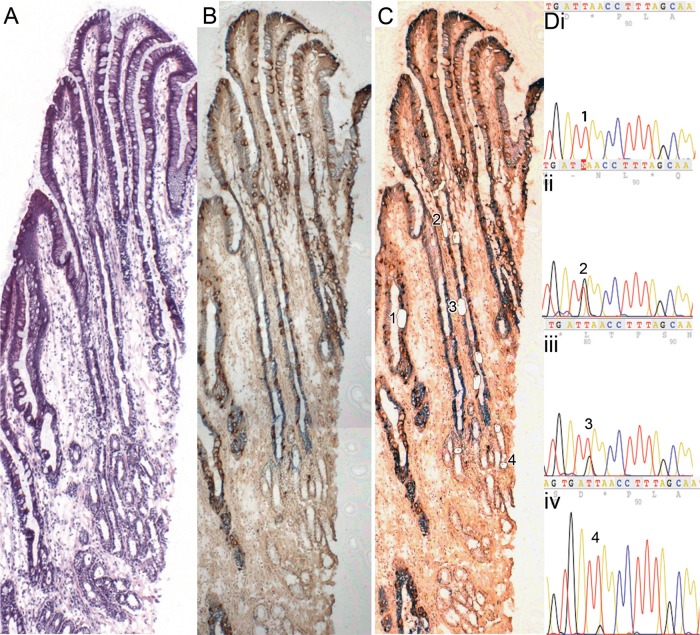
A well-orientated Barrett's gland. (A) An H&E; (B and C) stained with MUC5AC and MUC2 (figure 3B prelaser capture microdissection (LCM), figure 3C post-LCM). (D) Cells microdissected from the gland all contain the same heteroplasmic m.825 G>T mutation in the MT-RNR1 gene. MUC2 cells (i) wild-type cells; (ii) MUC2 cells, (iii) MUC5AC cells, (iv) basal mucous-secreting cells (note: a lower level of heteroplasmy was detected), (iv) all share this mutation, but cells from the neighbouring wild-type gland do not. Online supplementary figure S4 shows high power views of the cells dissected.

Figure 3 shows a well-orientated Barrett's gland (H&E figure 3A) stained with anti-MUC5AC and anti-MUC2 (figure 3B pre-laser capture microdissection (LCM), figure 3C post-LCM,): cells microdissected from the gland all contain the same heteroplasmic m.825 G>T mutation in the MT-RNR1 gene. MUC2+ cells (figure 3Dii), MUC5AC+ cells (figure 3Diii) and basal mucus-secreting cells (figure 3Div) all share this mutation, but cells from a neighbouring gland do not (figure 3Di). Online supplementary figure S4 shows high-power views of the cells dissected.

### Gene expression in Barrett's glands

**Figure 4:**
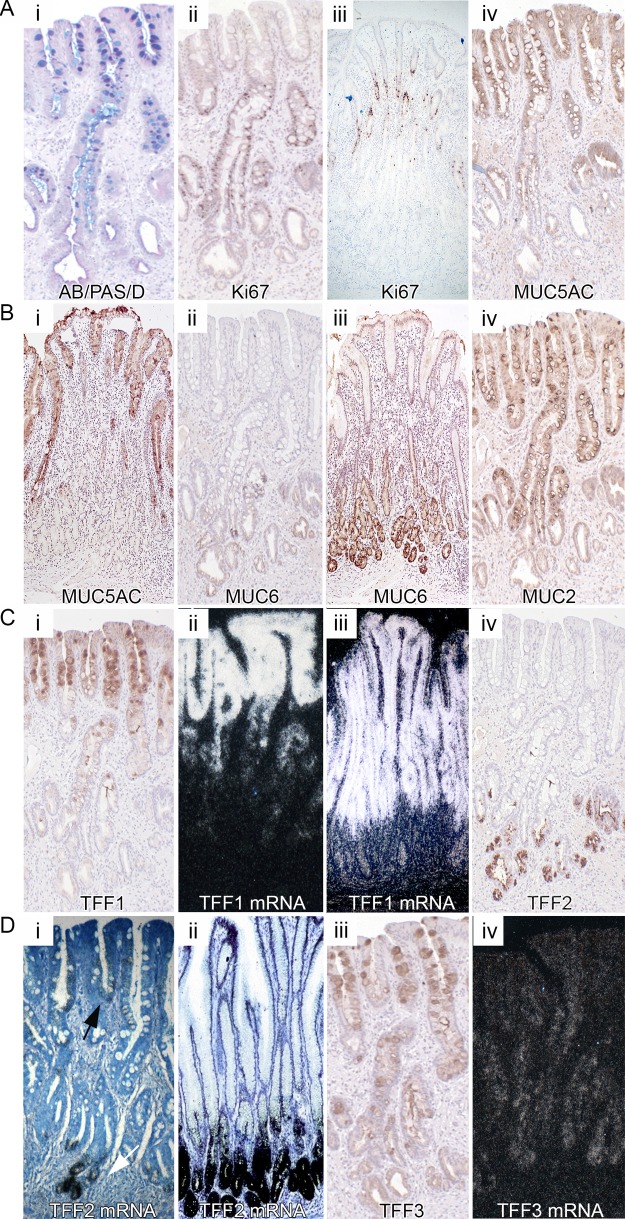
Gene expression in Barrett's glands compared with pyloric glands. Well-orientated glands displaying a contiguous surface, middle and base were analysed. (A) (i) Barrett's stained with D/PAS/Alcian Blue; (ii) Ki67 protein expression in Barrett's glands; (iii) in pyloric glands; (iv) MUC5AC protein expression in Barrett's glands. Figure 4B (i) MUC5AC protein expression in pyloric glands; (ii) MUC6 protein expression in Barrett's glands; (iii) in pyloric glands; (iv) MUC2 expression in Barrett's glands (see online supplementary figure S5A shows MUC2 to be absent from pyloric glands); figure 4C (i) TTF1 protein and (ii) mRNA expression in Barrett's glands; (iii) trefoil family factor 1 (TFF1) mRNA expression in pyloric glands. Supplementary figure 5B shows MUC5AC protein also in the upper part of pyloric glands; (iv) TTF2 protein in Barrett's glands. Figure 4D (i) mRNA expression in Barrett's glands; (ii) TFF2 mRNA in pyloric glands; (iii) TFF3 protein and (iv) mRNA in Barrett's glands.

Figure 4Ai shows that appropriately sectioned Barrett's glands appear slender and elegant, with a single surface opening or foveolus, and about half-way down the gland divides into a number of basal tubules, similar to the disposition of the gastric gland.^12^ The upper part of the gland contains columnar cells that stain with D/PAS and Alcian Blue, and there are also numbers of alcianophilic, sialomucin-containing goblet cells and non-goblet cells. In the base of the gland, there is an area, of variable size, which contains D/PAS+ mucous cells only. The proliferative zone (Ki67+) in this gland is seen above this area, extending into the upper part of the gland (figure 4Aii), similar to that seen in gastric glands (figure 4Aiii).

In Barrett's glands and pyloric glands, MUC5AC is seen exclusively in the upper part of the gland, decreasing in intensity as the stem cell zone, delineated by *LGR5* mRNA expression, is reached (figure 4Aiv and Bi respectively). MUC6 expression in Barrett's and pyloric glands is confined to the mucous cells at the base of the glands below the *LGR5* mRNA zone (figure 4Bii and iii). MUC2 shows a tight distribution throughout the upper part of the Barrett's gland, concentrated but not confined to the goblet cells, diminishes in expression towards the LGR5 zone, and is absent from the MUC6+ bases (figure 4Biv). MUC2 is absent from the pyloric epithelium (see online supplementary figure S5A). TFF1 protein and mRNA is located in the upper part of Barrett's and pyloric glands, coexpressed with MUC5AC (figure 4Ci, ii and iii, respectively, and see online supplementary figure S5B). TFF2 is seen confined largely to the MUC6+ cells in the base of Barrett's and pyloric glands (figure 4Civ, Di and ii, respectively, and see online supplementary figure S5C). *TFF2* mRNA is also present in low concentration in the foveolar equivalent of the Barrett's gland (figure 4Di, arrow). TFF3 protein is expressed throughout the Barrett's gland, concentrated in the goblet cells in the upper part of the gland (figure 4Diii), confirmed by the distribution of *TFF3* mRNA (figure 4Div). TFF3 is absent from the pyloric mucosa, unless intestinal metaplasia is present (see online supplementary figure 5Di and ii). In total, 15 patients with Barrett's metaplasia and 15 normal stomachs taken outside the resection margins of patients with gastric adenocarcinoma were used. All results described were observed in all samples.

The pattern of gene expression in antral or pyloric mucosa is thus reflected in Barrett's glands.

### Cell proliferation and gene expression in Barrett's dysplasia

**Figure 5:**
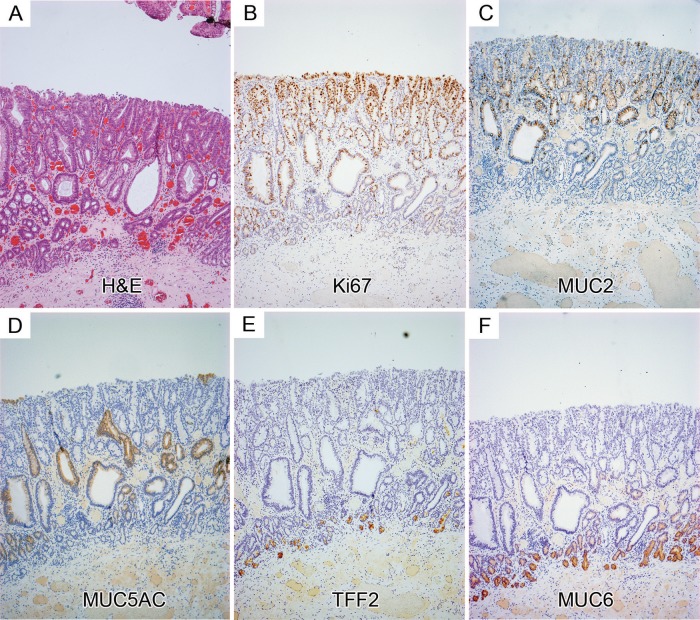
Protein expression in low-grade Barrett's dysplasia. (A) An H&E; (B) Ki67 expression: (C) MUC2 expression; (D) MUC5AC expression; trefoil family factor 2 (TFF2); (E) and MUC6 (F) colocalise in the mucous cell bases of the gland, which remain in dysplasia.

**Figure 6:**
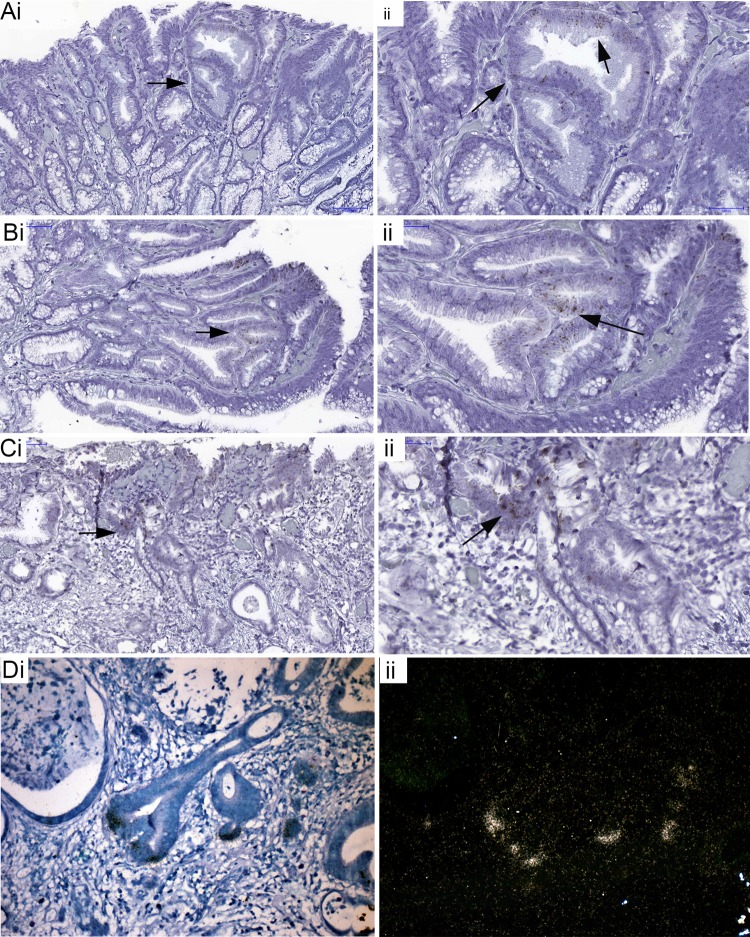
*LGR5* mRNA expression in Barrett's dysplasia and carcinoma. (A-C) low- (i) and high-power (ii) images showing non-isotopic ISH for *LGR5* mRNA localisation in Barrett's dysplasia; (D) (i) bright field image and accompanying dark field image (ii) of isotopic ISH for *LGR5* mRNA localisation in invasive Barrett's carcinoma glands.

Figure 5 shows the distribution of Ki67+ cells, mucin core proteins and TFFs in low-grade Barrett's dysplasia (figure 5A H&E): the proliferative organisation mirrors that seen in Barrett's glands and, thus, in gastric glands, with preservation of the non-proliferative MUC6+/TFF2+ cells at the base of the gland. Figure 5B shows that the proliferative zone indicated by Ki67 expression expands towards the top of the glands, while the bases remain largely non-proliferative. MUC2 is present in the middle and top of the glands (figure 5C) and MUC5AC on the surface and in the middle of glands (figure 5D): the basal TFF2+/MUC6+ mucous zone is preserved (figure 5E and F). *LGR5* mRNA+ cells are seen again above the MUC6+/TFF2+ cells, but there is some expansion of the zone towards the surface (figure 6Ai–Cii). These data are representative of 15 Barrett's dysplasia specimens. In Barrett's carcinoma (figure 6Di and ii) *LGR5* mRNA shows a specific localisation at the bases of the malignant glands (n=5).

### Gene expression in gastric intestinal metaplasia (IM)

In gastric intestinal metaplasia, especially in the so-called ‘mixed’, there is expression of gastric and intestinal mucins.^16^ Similarly, in partially intestinalised gastric glands, the distribution of the TTFs follows the pattern seen in Barrett's glands (see online supplementary figure S6). TFF2 and MUC6 (see online supplementary figure 6A, D) is seen at the bases, in continuity with goblet cell-containing intestinalised glands. *TFF1* mRNA is seen at the apices of such partially intestinalised glands (see online supplementary figure S6B), even where goblet cell differentiation is clearly seen (arrow). *TFF2* mRNA is concentrated in the gland base (see online supplementary figure S6C), although as in Barrett's glands (figure 4, white arrow), it is also seen in the goblet cell-containing surface (black arrow). *TFF3* mRNA is seen throughout the gland (figure 6E), but prominent in surface goblet cells (arrow). *LGR5* mRNA expression becomes localised in the isthmus/neck equivalent in dysplastic glands (see online supplementary figure S6Fi and ii), and the basal MUC6+/TFF2+ zone is preserved (arrowed).

## Discussion

The gland pattern of Barrett's glands is that of glands of the pyloric mucosa: the proliferative zone is housed within the isthmus/neck equivalent of the gland, and cell migration is bidirectional, upwards into the foveolus equivalent, and downwards into the tubules of the gland base. The stem cell zone, as indicated by the presence of *LGR5* mRNA+ cells, is located in the lower part of this area, almost at the level of the D/PAS+, TFF2+, MUC6+ cells (figure 4), while *LGR5* mRNA+ cells in the antral glands are seen mainly in the isthmus/neck. MUC5AC and TFF1 are found in the upper part of the gland, while TFF2 and MUC6 concentrated in the D/PAS cells at the gland base, as seen in antral gastric glands. MUC2, and to a lesser extent TFF3, is seen predominantly in goblet cells in the upper part of the gland. The organisation of Barrett's glands, their proliferative architecture, stem cell localisation and patterns of gene expression, directly reflects the pyloric gastric gland. We conclude that Barrett's glands contain equivalents of the foveolus, isthmus, neck and base seen in gastric glands.

The distribution of Ki67+ cells has been studied in Barrett's glands previously, and similar patterns seen to those described herein, persist even in dysplasia.^17^ The dynamic aspects of cell migration have not been studied: it should be recalled that Ki67 labels all cells in the cell cycle and the distribution seen in figure 1 reflects the distribution of cycling cells, effectively at time 0. IdU labels cells only in the S phase, and earlier readings available at 7 days would then reflect the position of cells labelled 7 days previously (note that the very basal gland is devoid of Ki67+ cells: see online supplementary figure S1). The fraction of IdU+ cells labelled in the foveolar region now equals that seen in the erstwhile proliferative zone. This means that cells have migrated, fairly rapidly, upwards to the foveolus. In the base, there are, at this time, relatively few labelled cells, indicating that migration to the base is slower. At 11 and 29 days, while labelled cells are lost from the foveolar region (by extrusion from the surface), and from the proliferative zone, labelled cells remain in the base, and by 67 days, only the base contains any labelled cells.

Migration in Barrett's glands is bidirectional: the detailed studies of Hattori and Fujita^18^ in the hamster corpus and pylorus have shown that, while cells migrate to the surface in some 14 days, over 300 days are needed to reach the corpus gland base. In humans, foveolar cells migrate to the surface rapidly (see online supplementary figure S1), but labelled parietal cells are seen in the isthmus at 67 days after IdU injection,^14^ indicating that in the human being, a similar lengthy period is needed for cells to reach the gland base, confirming bidirectional flux.

LGR5 has been shown to be a true stem cell marker in the mouse by lineage labelling.^15^ LGR5+ cells represent a stem-like cell population in colon carcinomas, which are also found in the bases of crypt-like structures within the tumour that resembles normal crypts^19^ (and see figure 6D). The presence of *LGR5+* cells and all differentiated lineages within colon carcinomas^20^ and adenomas has been reported,^19^ suggesting that LGR5 detects stem cell populations in human epithelia. *LGR5* mRNA localises specifically to the junction between the TFF1+/MUC5AC+/MUC2+ cells and the basal TFF2+/MUC6+ cells, and represents the stem cell zone, or niche, of Barrett's glands. Cells, including the MUC2+ cells destined to be goblet cells, migrate upwards from here, while the TFF2+/MUC6+ cells migrate downwards. In pyloric glands, *LGR5* is also localised in the isthmus/neck region, and a similar pattern of gene expression is seen. Contrast these findings with those exhibited in gastric intestinal metaplasia (figure 2E,F): *LGR5* mRNA is seen in the base, as in the small intestinal crypt (see online supplementary figure S3) and colonic crypts.^19^ Barrett's carcinoma glands show *LGR5* mRNA localisation very similar to that described by Merlos-Suarez *et al*^20^ (ISH was used as IHC for human LGR5 is unreliable) (figure 6D).

There have been previous studies of the distribution of mucin gene expression^6^ ^21^ and also of TFFs^22^ ^23^ in Barrett's glands: selective segregation of MUC5AC with TFF1 and MUC6 with TFF2 is seen in Barrett's glands, mirroring antral gastric glands.^24^ TFF3 is usually colocalised with MUC2, but in Barrett's glands, it is expressed in the MUC6+/TFF2+ basal mucous cells, differing from antral glands and also in incompletely intestinalised intestinal metaplastic glands (see online supplementary figure S6E). TFF3 expression has been described in the normal human stomach,^25^ although in our hands not unless intestinal metaplasia is present (see online supplementary figure S5D). *TFF2* mRNA is also expressed, in low concentration, in the ‘foveola’ of Barrett's and partially intestinalised gastric glands (see figures 4Di, ii and also online supplementary figure S6C).

An intriguing point is the relationship of Barrett's metaplasia to intestinal metaplasia.^26^ ‘Mixed’ intestinal metaplasia occurs in the stomach,^16^ and online supplementary figure S6 shows that such mixed types of intestinal metaplasia show a similar pattern of TFF expression as Barrett's glands—the mucin histochemical profiles also show a mixed pattern,^16^ suggesting that Barrett's glands resemble the mixed metaplastic glands seen in the stomach.

**Figure 7:**
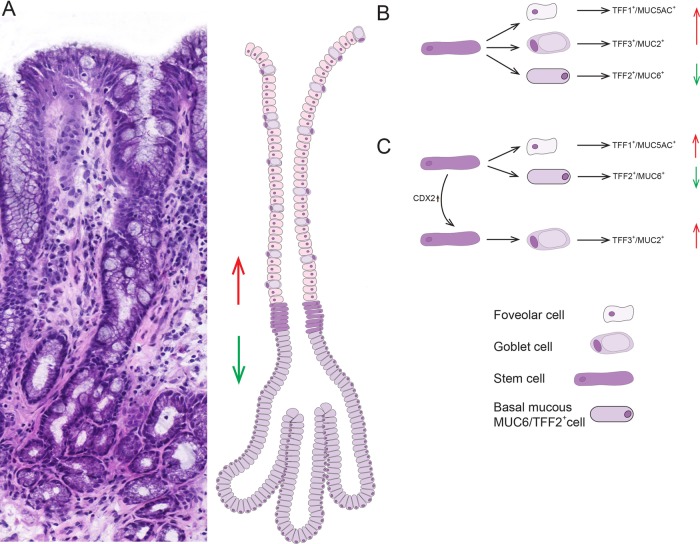
(A) An H&E of a well-orientated Barrett's glands with diagrammatic representation of a model of organisation in Barrett's glands; the stem cell zone, here visualised as a ring of 6–7 cells, occupies the centre of the gland immediately above the point of branching. The trefoil family factor 1 (TFF1)+/MUC5AC+/MUC2+ cells migrate upwards from this zone while the TFF2+/MUC6+ cells migrate towards the base. (B, C) Possible models for stem/committed progenitor lineage relationships in Barrett's glands. Two possibilities are shown: (B) where a single stem cell gives rise to committed progenitors for the TFF1+/MUC5AC+ cells, the TFF3+/MUC2+ cells and the TFF2+/MUC6+ cells. (C) A neutral drift model where there are stem cells which produce TFF2+/MUC6+ cells, and stem cells which produce TFF1+/MUC5AC+ cells: following an event such as activation of CDX2, this stem cell(s) commit to produce TFF3+/MUC2+ cells, and stochastic niche succession will eventually, in some glands, move entirely to a niche containing stem cells committed to the TFF3+/MUC2+ lineage. We propose a conversion from non-goblet containing columnar to a specialised epithelium and finally to intestinal metaplasia.

Barrett's glands are clonal populations^13^ (figure 3). Established Barrett's glands contain multiple cell lineages, MUC5AC+/TFF1+, MUC2+ and MUC6+/TFF2+, and in Barrett’s epithelium, gastric and intestinal endocrine cells are found.^27^ The stem cell niche in the isthmus/neck equivalent area of the gland contains stem cells capable of delivering all these lineages. Either the niche contains unique stem cells capable of giving rise to all contained lineages, or the niche is composed of a mixture of stem cells with limited repertoire, for example, limited to TFF1+/MUC5AC+ and TFF2+/MUC6+ lineages, and other stem cells give rise to the TFF3+/MUC2+ lineage (figure 7).

The implication here is that Barrett's glands were originally gastric glands containing TFF1+/MUC5AC+ and TFF2+/MUC6+ lineages. The homeobox gene, CDX2, is associated with activation of intestinal differentiation.^2^ This occurs in the stomach as a result of *Helicobacter pylori* infection, and could occur in Barrett's glands as a result of continued bile/acid refluxate. CDX2 expression in a stem cell will induce progenitors of the TFF3+/MUC2+ lineage. Most stem cell divisions are symmetrical, and neutral drift governs stem cell dynamics:^28^ stochastically, a TFF3+/MUC2+ stem cells may survive in the niche, and the gland then contains the first stem cell committed to intestinal differentiation. Neutral drift dynamics would predict that such a stem cell could be lost, and the gland maintains its gastric phenotype, but the stem cell could expand in the niche, giving the Barrett's phenotype of mixed gastric and intestinal lineages. Eventually, niche succession would fill the niche with MUC2+/TFF3+ stem cells, giving a fully intestinalised gland, seen in Barrett's oesophagus.^9^ ^10^ We would state that causality cannot be inferred from the observational data provided. Gastric intestinal metaplasia may also go through such a transitional phase^16^ (see online supplementary figure S6). Cells expressing gastric and intestinal markers are seen, possibly the progeny of stem/progenitor cells where CDX2 is only partially activated, and where gastric and intestinal gene expression coexists.

The gland pattern, including the basal TFF2+/MUC6+ cells, are preserved in dysplasia, with dysplastic cells confined to the upper part of the gland; in gastric carcinoma, the dysplastic cells may originate in the deep pit or isthmus of the gastric gland:^29^ in Barrett's oesophagus, the dysplastic part of the gland is clonal for p16 mutations even when the surface cells lack dysplastic features.^30^ It is not yet known if this is true also for the basal TFF2+/MUC6+ basal mucous cells. Dysplasia and indeed carcinoma can show gastric and intestinal differentiation markers.^7^ Dysplasia may arise through mutational events in stem cells: hence, dysplasia developing in TFF1+/MUC5AC+ or in TTF3+/MUC2+ committed stem/progenitor cells may account for the origin of the dysplasia in the stem cell zone and also for the several differentiation patterns described.^7^

Barrett's segments may develop from upward progression of the cardiac mucosa, a concept which appears to have fallen out of favour in recent years in favour of a metaplastic origin from oesophageal squamous epithelium.^2^ But there is older evidence, such as the development of neo-Barrett's after subtotal oesophagectomy and reconstruction with a gastric conduit: about 50% of these patients develop columnar epithelium in the area lined by squamous mucosa at the time of the procedure, and the length of columnar mucosa increases with longer follow-up.^31^ Recent evidence from animal models,^1^ and from following the progression of metaplasia at the cardia,^32^ is supportive of such a proposal. Whichever theory is entertained, it will have to account for the observation that Barrett's glands replicate the organisation of gastric glands.

## Supplementary Material

Web supplement

Web supplement
