# Supplementary material for: The stem cell organisation, and the proliferative and gene expression profile of Barrett's epithelium, replicates pyloric-type gastric glands
Source: Gut. 2014 Feb 18;63(12):1854–63. doi: 10.1136/gutjnl-2013-306508 (PMC4251192; doi:10.1136/gutjnl-2013-306508)
Supplement: Web supplement [file gutjnl-2013-306508-s1.pdf]

Supplementary Methods

Immunohistochemistry (IHC):

Five µm FFPE tissue sections were dewaxed in xylene and hydrated through a graded ethanol series to water. Antigen retrieval was performed in boiling tris-EDTA buffer solution, pH 9.0 (Sigma, Poole, Dorset, UK) for 15 minutes. Endogenous peroxidase activity was blocked with 3% hydrogen peroxide solution for 10 minutes, followed by a second block in tris-buffered saline (TBS) with 5% donkey serum, 0.5% skimmed milk powder, 0.25% fish skin gelatin and 0.1% tween for 30 minutes.

Sections were incubated with primary antibodies for Ki67, MUC2, MUC5AC, MUC6, TFF1, TFF2 and TFF3 overnight at 4°C. Details are outlined in Supplementary Table 1. All antibodies were diluted in TBS/5% donkey serum and TBS/5% donkey serum alone was substituted for each primary antibody on all tissues as a negative control. For the sections incubated with mucin or trefoil family factor primary antibodies, the second layer was horseradish peroxidase (HRP) conjugated goat anti-rabbit or goat anti-mouse IgG (Sigma A0545 and A0412, respectively) added at a 1:100 dilution and incubated for one hour at room temperature.

For the sections incubated with Ki67 primary antibody, the second layer was biotin conjugated goat anti rabbit IgG (Dako, Ely, Cambridgeshire, UK; E0432) added at a 1:400 dilution and incubated for one hour at room temperature. A third layer of streptavidin conjugated HRP at a 1:500 dilution was added for 30 minutes at room temperature (Dako, P0397). A brown reaction product was obtained for all sections using 0.6 mg/ml 3,3'diamino-benzidine (DAB) with 0.03% (v/v) H_2O_2 (Dako) and sections were counterstained with haematoxylin, before dehydration and mounting.

Double IHC for MUC2 and MUC5AC:

Ten μm frozen tissue sections were cut serially onto laser capture microdissection (LCM) slides (Zeiss, Munich, Germany) and fixed in 4% paraformaldehyde for 5 minutes at room temperature, before blocking as described in the IHC protocol above. Sections were incubated with primary antibodies for MUC2 and MUC5AC together overnight at 4°C (Supplementary Table 1). Sections were incubated with alkaline phosphatase conjugated sheep anti-mouse IgG (Sigma, A3563) for one hour at room temperature. A blue reaction product representing MUC5AC expression was obtained using a Vector blue substrate kit (Vector Labs Ltd, Peterborough, Cambridgeshire, UK). Sections were then incubated with HRP conjugated goat anti rabbit IgG for one hour at room temperature, and a brown reaction product representing MUC2 expression was obtained using DAB as described above. Sections were dehydrated through a graded ethanol series, cleared in histoclear (Lamb Laboratory Supplies, Eastbourne, UK) and then allowed to dry before microdissection with a P.A.L.M. laser capture microscope (Zeiss).

PCR sequencing of MtDNA from laser capture samples

Microdissected cells were digested in 14 ml Picopure (Life Technologies, UK) at 65ºC for 3 hours then denatured at 95ºC for 5 mins. A nested PCR protocol was followed as per previously described[12,13]. Briefly the mitochondrial genome was amplified into nine 2 kb fragments, which were then subsequently amplified into 500 bp fragments. Primer sequences and PCR conditions were as previously[12,13]. The second round PCR primers contained an M13 sequence to facilitate sanger sequencing. PCR products were ExoSaP-IT treated according to manufacturers protocol (GE Healthcare UK) and subjected to a sequencing reaction using Big Dye 3.1 (Applied biosystems, UK) and run on a ABI Prism 3100 genetic analyser (Applied Biosystems). Obtained sequences were viewed using 4Peaks software (www.mekentosj.com) and compared to the revised Cambridge reference sequence (www.mitomap.com). Polymorphisms were eliminated from analysis by comparing the obtained sequence from a cell distant to experimental cells and comparing to know polymorphisms (www.mitomap.com). PCR sequencing was repeated from the original DNA sample.

Isotopic in situ hybridization (ISH):

Sections were labeled with riboprobes for human (h) TFF1-3, GKN2 (full-length IMAGE clone 1693411) and compared to control sections labeled with a riboprobe for [beta]-actin (ACTB). 35S-labeled riboprobes were prepared on 5 µm tissue sections as previously described [w1]. Autoradiographs were exposed for seven days and up to 14 days depending on the first signal intensity and were counterstained with Giemsa. Sections were examined with transmitted light (bright-field) and epi-illumination (dark-field) using a Nikon Eclipse ME600 microscope (Nikon UK Ltd, Kingston upon Thames, UK) and a digital camera (Q Imaging MicroPublisher 5.0 RTV, Surrey, British Columbia, Canada).

For the other trefoil family factors we used a TFF1 sequence corresponding to nucleotides 1–408 of human TFF1, an approximately 300 bp sequence for TFF2 and a 221 bp sequence for TFF3. For LGR5, ISH was carried out with 3H or 35S antisense riboprobes essentially as described[33] with SP6 RNA polymerase and EcoRI linearized sequence verified templates prepared in pGEM3Z by Dr Stefania Segditsas for human LGR5 566 bp from 5′UTR to exon5 (UCSC chr12: 70,120,102-70,233,231, introns excluded).

Non-radioactive in situ hybridization (NISH)

LGR5 mRNA was localised in 5 µm sections of formalin fixed paraffin-embedded tissue by using an RNAscope 2.0 Brown kit and probes purchased from Advanced Cell Diagnostics Inc (Hayward, CA www.acdbio.com) according to the manufacturer's protocol. Discrete small brown signals were produced in similar locations when either 1:5 or 1:15 dilution of the kit protease was used. Positive control probes included POL2RA and PPIB, with DapB used as a negative control probe. *LGR5* mRNA expression as visualized by RNAscope 2.0 was broadly similar to that seen using 35S riboprobe ISH on sections from a number of test blocks.

Clinical protocol for labelling with iododeoxyuridine (IdU):

Two sites were used for tissue acquisition: Gloucestershire Royal Hospital and Leicester Royal Infirmary, both in England. Four patients were recruited to the study. After informed consent was obtained an intravenous infusion of 5-iodo-2'-deoxyuridine (IdU) at a dose of 200 mg per m^2 body surface area (maximum dose of 400 mg) was given over a 30-minute period. Each 200 mg vial was reconstituted with 10 ml of water and the resultant solution was then added to 250 ml of 0.9% sodium chloride to generate the infusion solution. Following the infusion the vital signs were recorded and patients were monitored every 30 minutes for a further three hours.

Patients 1 and 2 underwent esophagectomy seven days post-infusion. Patient 3 was infused 11 days prior to surgery; Patient 4 was infused 29 days prior to an esophageal endoscopic mucosal resection (EMR) for dysplasia and an esophagectomy for esophageal adenocarcinoma, 67 days post-infusion. None of the patients underwent pre-operative chemo-radiotherapy. Tissues were obtained from areas of Barrett's and from normal stomach within the resection margins.

IdU+ cells were detected on 5 µm tissue sections following antigen retrieval in 2 M hydrochloric acid for 30 minutes at 37°C. Sections were neutralised in 0.1 M Borax (Sigma) for five minutes before blocking as per the IHC protocol and then incubated with anti-BrdU primary antibody at 4°C overnight (Supplementary Table 1). The secondary layer was a biotin conjugated rabbit anti-mouse antibody (Dako, E0354) added at a 1:300 dilution and incubated for one hour at room temperature. A third layer of streptavidin-conjugated HRP was then added and brown signal developed using DAB as for the IHC protocol.

Supplementary Table 1.

**Antibody**	**Details***	**Source**	**Host**	**Dilution**
TFF1	pAb, HPA003425	Sigma	Rabbit	1:1000
TFF2	NCL-HSP, clone GE16C, IgM	Leica	Mouse	1:25
TFF3	pAb antiserum	A gift of Dr Andy	Rabbit	1:1000

		Giraud, Melbourne, Australia		
MUC2	pAb, ab76774	Abcam	Rabbit	1:100
MUC5AC	NCL-MUC-5AC, clone 2b4, IgG1	Novocastra	Mouse	1:50
MUC6	NCL-MUC-6 Clone CLH5, IgG1	Novocastra	Mouse	1:25
Ki67	pAb, NCL-Ki67p	Novocastra	Rabbit	1:200
IdU	M0744, clone Bu20a, IgG1	Dako	Mouse	1:20

*pAb = Polyclonal antibody

Supplementary reference

W1. Poulsom, J.M. Longcroft, R.E. Jeffery, L.A. Rogers, J.H. Steel. A robust method for isotopic riboprobe in situ hybridization to localize mRNAs in routine pathology specimens. Eur. J. Histochem., 42 (1998), pp. 121–132

Supplementary figures

Supplementary Figure 1. Low (A, *denotes area of interest) and high-power (B) images of the bases of Barrett's glands showing that they are largely devoid of Ki67+ cells.

Supplementary Figure 2. (A, B) The distribution of Ki67+ cells in corpus gastric glands, showing most labelled cells with the isthmus/neck (C) IdU+ cells in gastric glands at 7 days post injection; (D) the distribution of IUdR+ cells at 7 days. (E) IUd+ cells in gastric glands at 11 days post injection; (F) the distribution of IdU+ cells at 11 days post-injection: at 7 days most IdU+ cells were identified within the foveolus of the gastric unit, although several are seen within the neck. At 11 days most IdU+ cells were lost from the epithelium and although some remain within the foveolus, the highest percentage was identified within the neck of the gastric unit.

Supplementary Figure 3. (A) A bright field image and (B) accompanying dark field image of *LGR5* mRNA sited at the bases of human colonic crypts.

Supplementary Figure 4. The gland microdissected in Figure 3 stained with MUC5AC and MUC2. High power view of (A) pre-LCM and (B) and C post-LCM respectively).

Supplementary Figure 5. (A) Showing absence of MUC2 staining in pyloric mucosa; (B) TFF1 protein expression in pyloric mucosa, showing localization in the upper part of the gland, as seen in Barrett's glands; (C) TFF2 protein expression in pyloric glands, showing localization in the bases of the glands, as seen in Barrett's glands: (D) ISH in gastric mucosa, showing *TFF3* mRNA expression only in an area of intestinal metaplasia (arrowed).

Supplementary Figure 6. The distribution of the trefoil family factors in partially intestinalized gastric glands follows the pattern seen in Barrett's glands. (A, D) shows TFF2 and MUC6 protein at the bases, in continuity with goblet cell-containing intestinalized glands. (B) Shows *TFF1* mRNA at the apices of such partially-intestinalized glands, present even where goblet cell differentiation is clearly seen

(arrow). (C): *TFF2* mRNA is heavily concentrated in the gland base, although as in Barrett's glands (Figure 4J) it is also seen in the goblet-cell containing surface (arrow). *TFF3* mRNA is seen throughout the gland (E), but prominent in surface goblet cells (arrow). It is interesting to see that, in gastric dysplasia, *LGR5* mRNA expression becomes localized (arrowed) in the isthmus/neck equivalent of the dysplastic gland (Fi and ii, light and dark-field).
