# Supplementary material for: The stem cell organisation, and the proliferative and gene expression profile of Barrett's epithelium, replicates pyloric-type gastric glands
Source: Gut. 2014 Feb 18;63(12):1854–63. doi: 10.1136/gutjnl-2013-306508 (PMC4251192; doi:10.1136/gutjnl-2013-306508)
Supplement: Web supplement [file gutjnl-2013-306508-s2.pdf]

Supplementary Figures

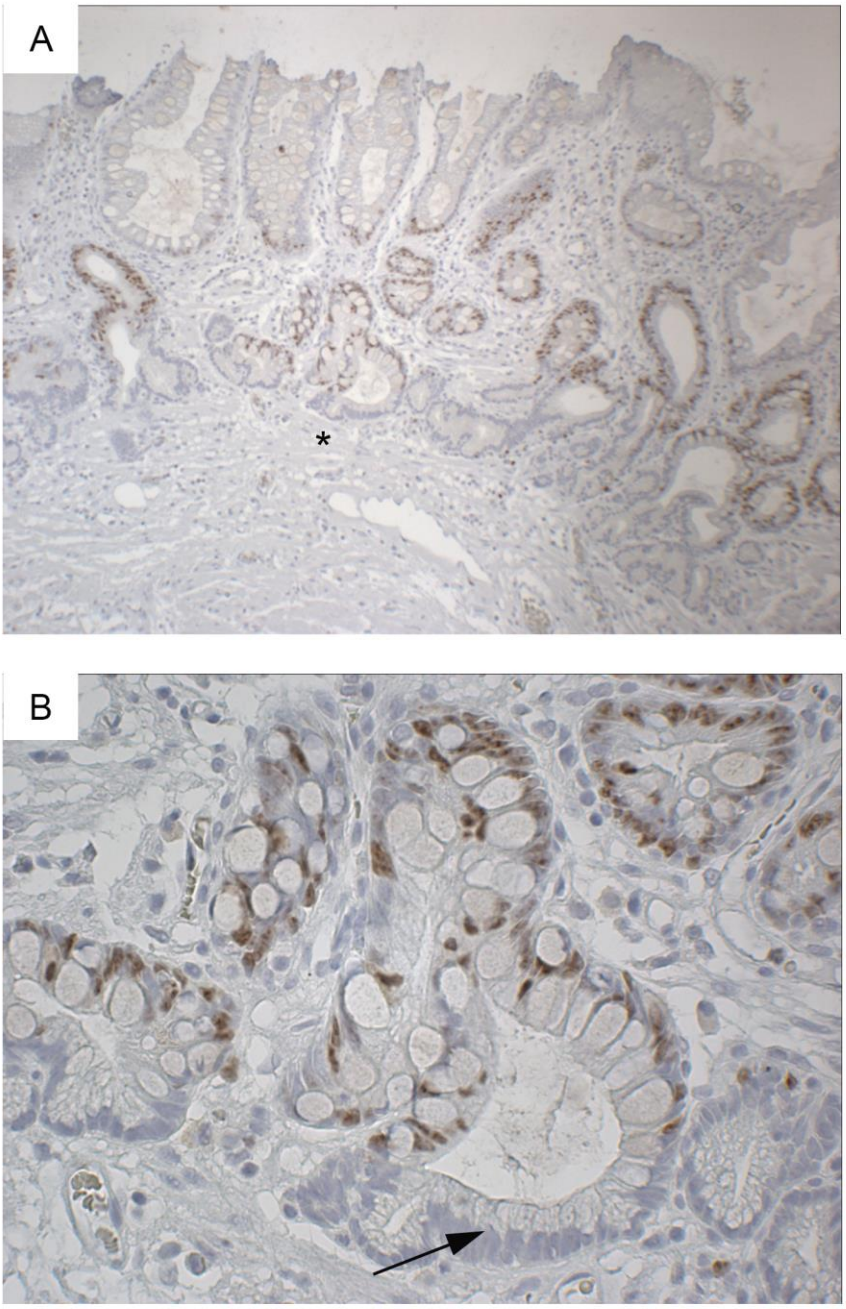

Supplementary Figure 1.

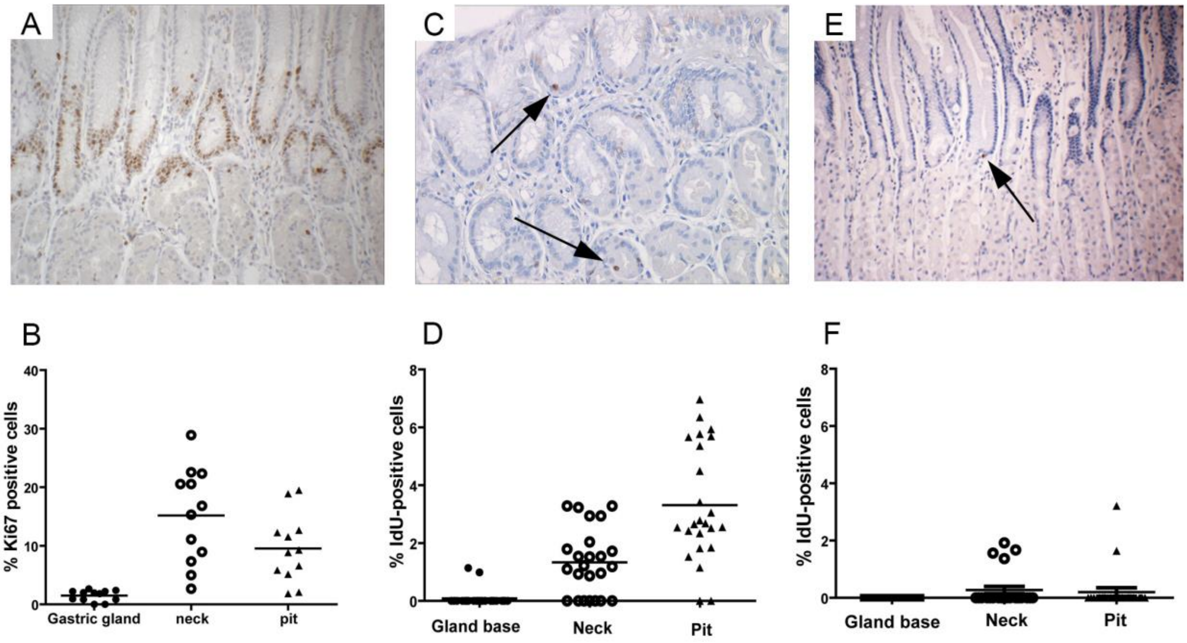

Supplementary Figure 2.

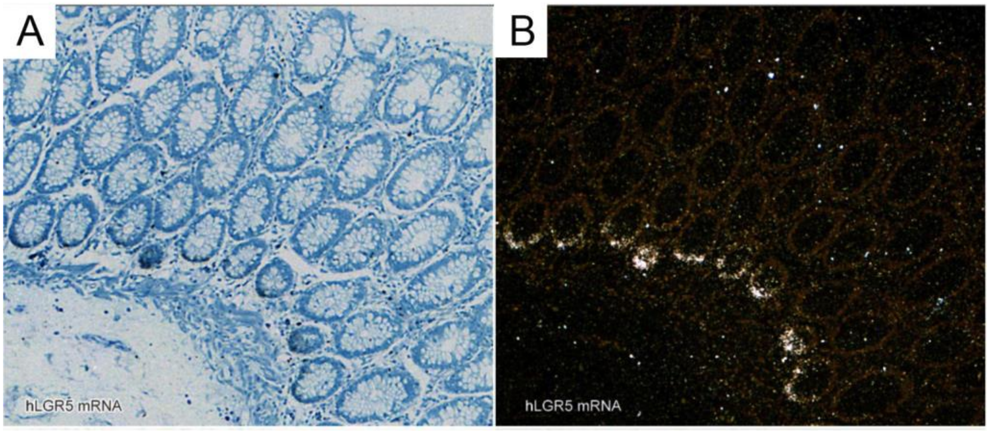

Supplementary Figure 3.

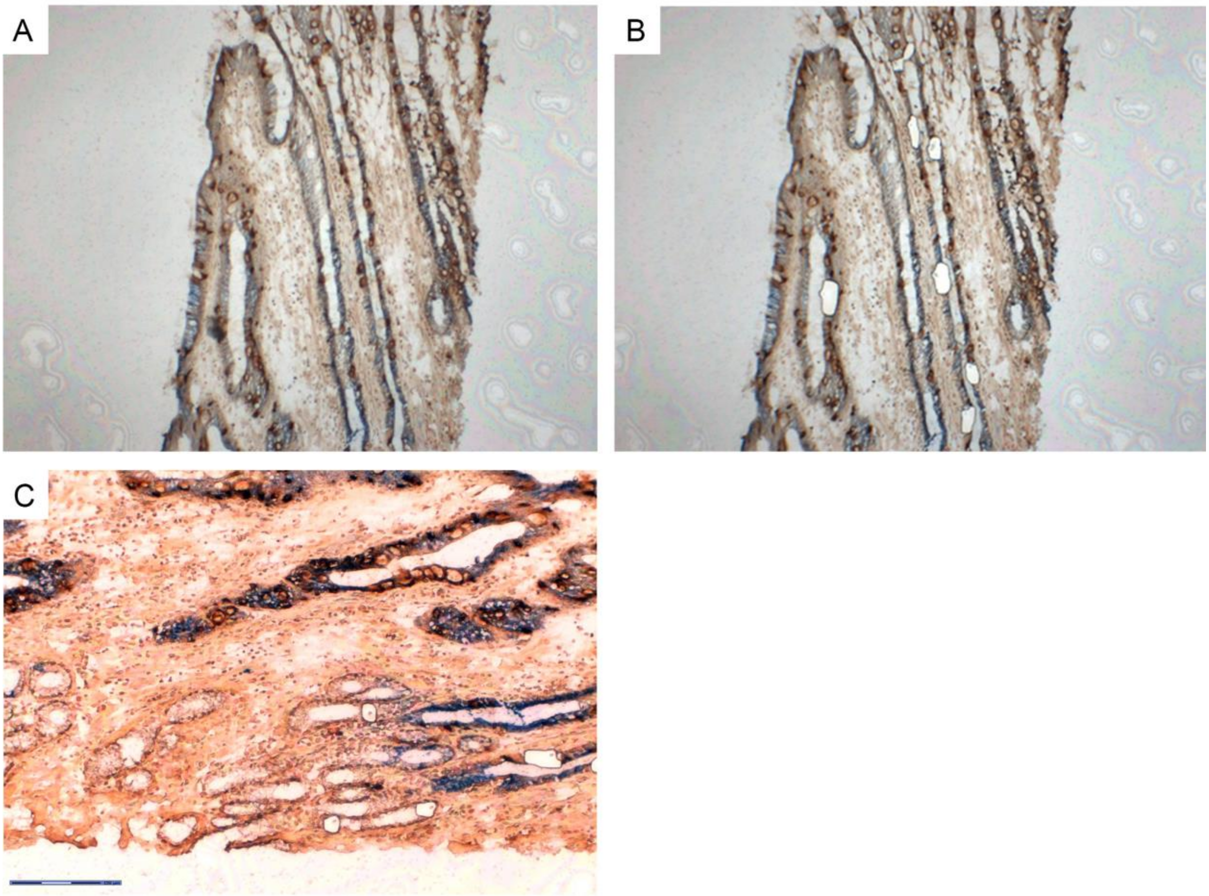

Supplementary Figure 4.

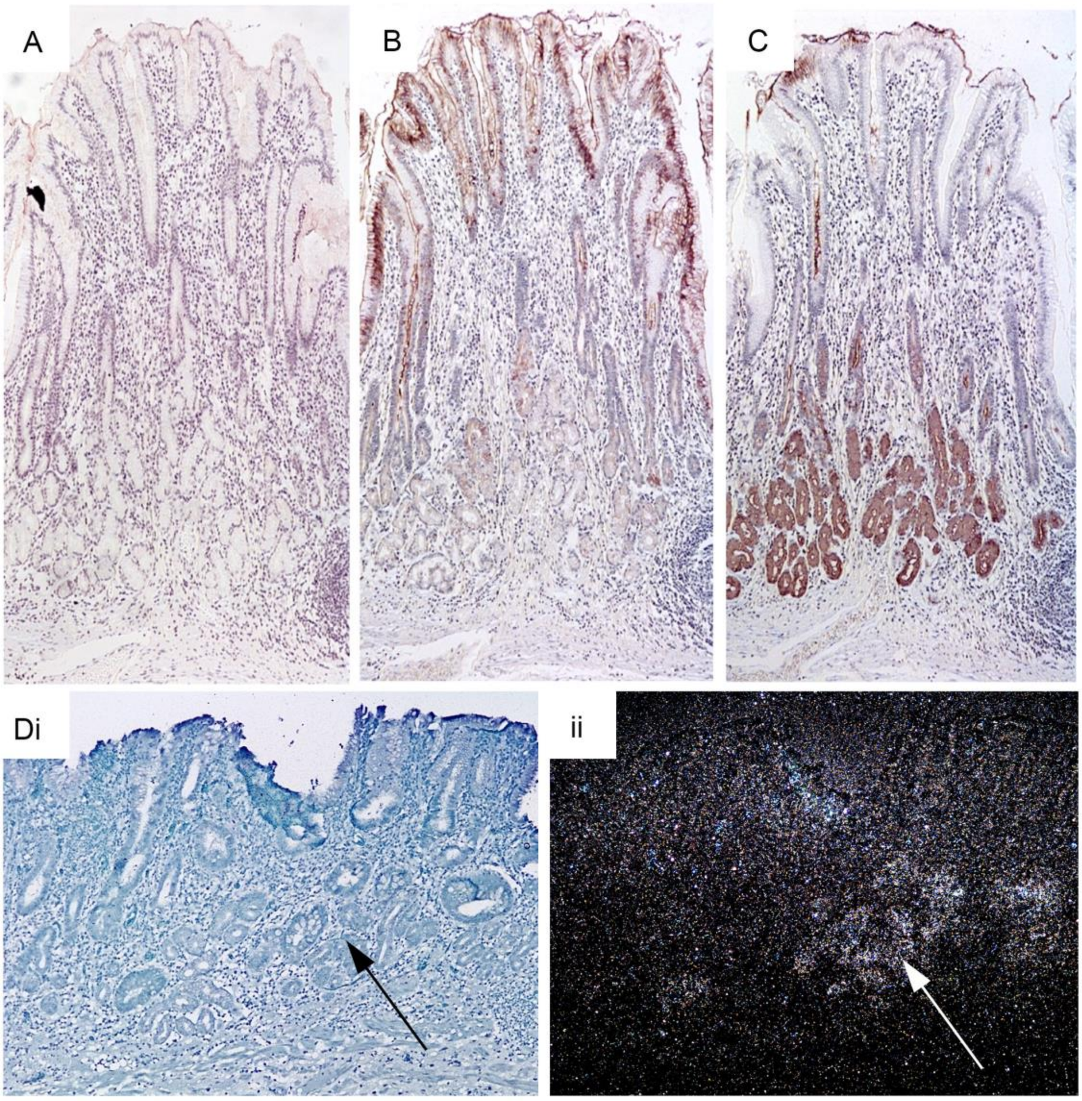

Supplementary Figure 5.

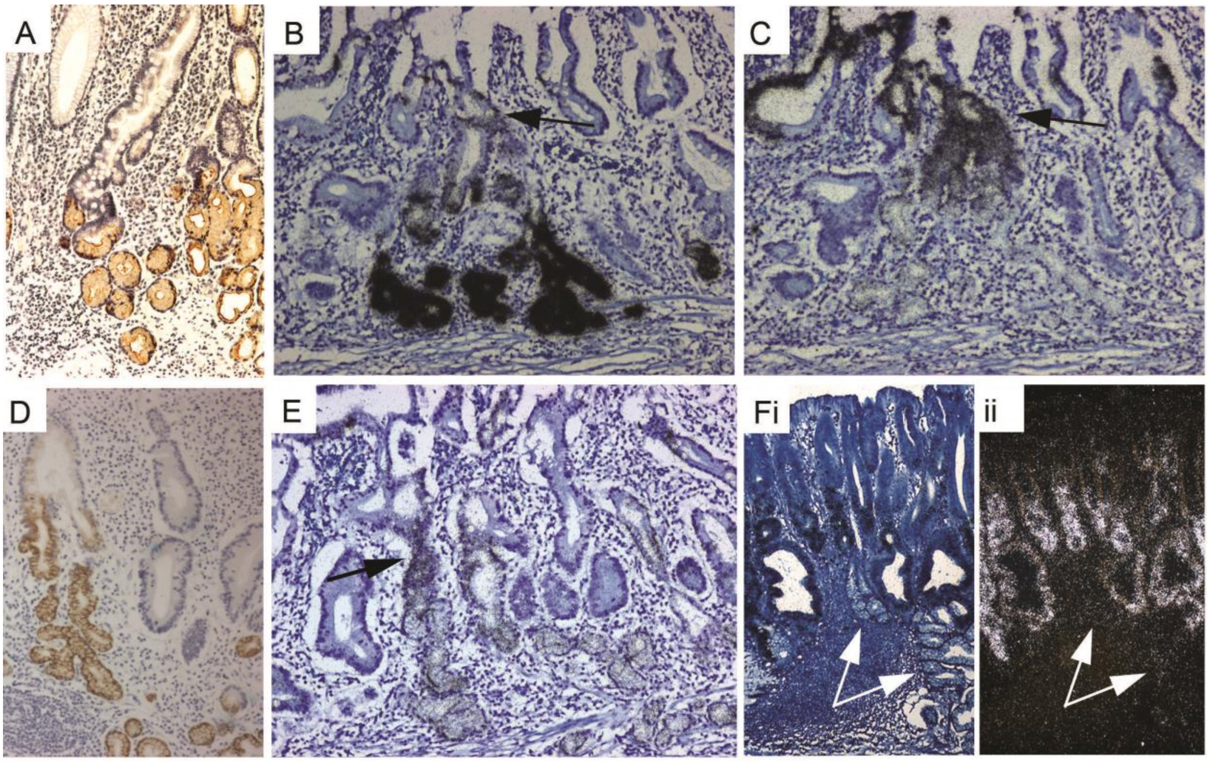

Supplementary Figure 6.
